# Transactions of the College of Physicians of Philadelphia

**Published:** 1877-10-20

**Authors:** 


Transactions of the College of Physicians of Philadelphia—Vol. 10th, 212 pages, neatly bound in muslin—containing a number of valuable papers.

Retarded Dilatation of the Os Uteri in Labor. —By Albert H. Smith, M. D., Philadelphia.

GOOD LOCATION FOR SALE.—Dr. Calfee, of Arkansas, offers a desirable property and practice. See his advertisement in this number.

